# Supplementary figures and images for: Biochemical composition, β-glucan and phenolic content of a marine diatom Chaetoceros muelleri cultivated in Guillard’s modified medium
Source: PeerJ. 2025 Sep 30;13:e20098. doi: 10.7717/peerj.20098 (PMC12493710; doi:10.7717/peerj.20098)

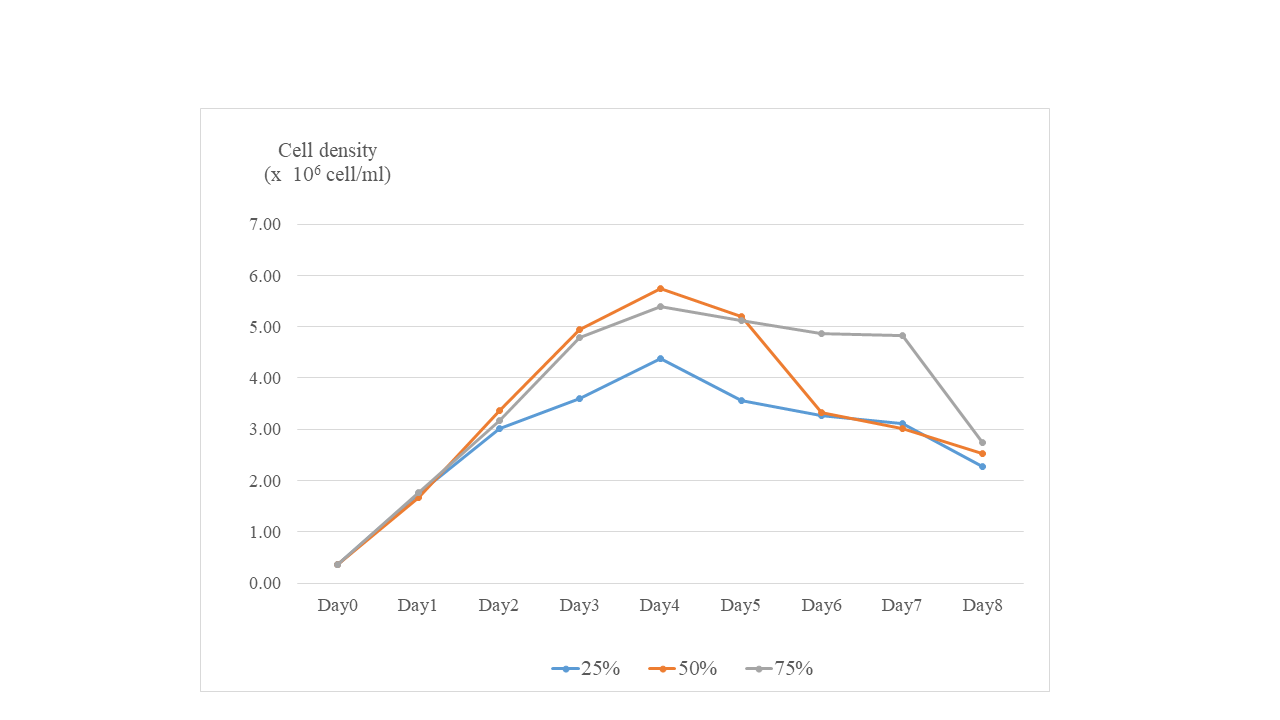

Supplement: Supplemental Information 12 [file peerj-13-20098-s012.png]
