# Supplementary material for: Biochemical composition, β-glucan and phenolic content of a marine diatom Chaetoceros muelleri cultivated in Guillard’s modified medium
Source: PeerJ. 2025 Sep 30;13:e20098. doi: 10.7717/peerj.20098 (PMC12493710; doi:10.7717/peerj.20098)
Supplement: Supplemental Information 13 — The mean and standard deviation (SD) of three replicates were used to express data. Different letters represent the statistical significant different at 95 % confident interval (p < 0.05) . [file peerj-13-20098-s013.pdf]

**Yield and biochemical composition**	**25%**	**50%**	**75%**
Biomass ($\times 10^6$ cells mL^{-1})	4.38±0.31^a	5.75±0.10^c	5.38±0.06^b
Yield of crude beta glucan (g L^1)	0.18±0.03^a	0.41±0.01^c	0.32±0.03^b
Total glucan (% w/w) (Megazyme analysis)	10.20±0.36^a	79.47±1.40^c	18.30±0.31^b
α-glucan (% w/w) (Megazyme analysis)	0.19±0.01^b	0.01±0.00^a	0.28±0.00^c
β-glucan content (% w/w) (Megazyme analysis)	10.01±0.36^a	79.46±1.40^c	18.02±0.32^b
