# Supplementary material for: Biochemical composition, β-glucan and phenolic content of a marine diatom Chaetoceros muelleri cultivated in Guillard’s modified medium
Source: PeerJ. 2025 Sep 30;13:e20098. doi: 10.7717/peerj.20098 (PMC12493710; doi:10.7717/peerj.20098)
Supplement: Supplemental Information 14 — The mean and standard deviation (SD) of three replicates were used to express data. Different letters represent the statistical significant different at 95 % confident interval (p < 0.05) . T1= standard Guillard F/2 medium; T2 = modified Guillard F/2 medium supplemented with 0.05 g L⁻¹ sodium bicarbonate; T3 = modified Guillard F/2 medium with a 50% reduction in nitrogen. [file peerj-13-20098-s014.docx]

**Table 1** Biomass of *C. müelleri* cultivation and yield extracted and biochemical composition of crude diatom beta glucan extracted from *C. müelleri*

| **Yield and biochemical composition** | **T1** | **T2** | **T3** |
| --- | --- | --- | --- |
| Biomass (x10⁶ cells mL^-1^) | 3.85 ± 0.13^a^ | 4.03 ±0.08^a^ | 5.75±0.06^b^ |
| The yield of crude beta-glucan extracted (g L^-1^) | 0.38±0.01^a^ | 0.35±0.01^b^ | 0.41±0.01^c^ |
| Carbohydrate (%) | 5.81 ±0.50^a^ | 6.90 ±1.14^a^ | 13.71± 1.56^b^ |
| Total proteins (mg/g extract) | 94.84±0.08^b^ | 31.39 ±0.72^a^ | 30.91 ±0.38^a^ |
| Lipid (%) | 1.1+0.08^a^ | 2.21+0.62^b^ | 4.34+0.03^c^ |

The mean and standard deviation (SD) of three replicates is used to express data. When employing one-way ANOVA (P< 0.05), different letters represent the statistically significant.

**raw data**

| **Biomass (x 10⁶ cells mL-¹)** | **T1** | **T2** | **T3** |
| --- | --- | --- | --- |
| R1 | 3.90 | 4.03 | 5.81 |
| R2 | 3.70 | 4.10 | 5.70 |
| R3 | 3.95 | 3.95 | 5.75 |
| **Average** | 3.85 | 4.03 | 5.75 |
| **SD** | 0.13 | 0.08 | 0.06 |

| **The yield of crude beta-glucan extracted (g L^-1^)** | **T1** | **T2** | **T3** |
| --- | --- | --- | --- |
| R1 | 0.38 | 0.35 | 0.41 |
| R2 | 0.39 | 0.34 | 0.42 |
| R3 | 0.38 | 0.36 | 0.40 |
| **Average** | 0.38 | 0.35 | 0.41 |
| **SD** | 0.01 | 0.01 | 0.01 |

| **Carbohydrate (%)** | **T1** | | **T2** | | **T3** | |  |
| --- | --- | --- | --- | --- | --- | --- | --- |
| R1 | 5.24 | | 5.76 | | 12.00 | |  |
| R2 | 6.19 | | 8.05 | | 14.10 | |  |
| R3 | 6.00 | | 6.90 | | 15.05 | |  |
| **Average** | **5.81** | | **6.90** | | **13.71** | |  |
| **SD** | 0.50 | | 1.14 | | 1.56 | |  |
| **Total proteins (mg/g extract)** | | **T1** | | **T2** | | **T3** | |
| R1 | | 94.75 | | 31.05 | | 31.34 | |
| R2 | | 94.89 | | 30.91 | | 30.76 | |
| R3 | | 94.89 | | 32.21 | | 30.62 | |
| **Average** | | **94.84** | | **31.39** | | **30.91** | |
| **SD** | | **0.08** | | **0.72** | | **0.38** | |

|  |
| --- |
| \| **Lipid (%)** \| **T1** \| **T2** \| **T3** \| \| --- \| --- \| --- \| --- \| \| R1 \| 0.99 \| 2.24 \| 4.32 \| \| R2 \| 1.09 \| 2.09 \| 4.37 \| \| R3 \| 1.25 \| 2,30 \| 4.34 \| \| **Average** \| **1.11** \| **2.21** \| **4.34** \| \| **SD** \| **0.08** \| **0.62** \| **0.03** \| |
|  |
