# Supplementary material for: Biochemical composition, β-glucan and phenolic content of a marine diatom Chaetoceros muelleri cultivated in Guillard’s modified medium
Source: PeerJ. 2025 Sep 30;13:e20098. doi: 10.7717/peerj.20098 (PMC12493710; doi:10.7717/peerj.20098)
Supplement: Supplemental Information 15 — The mean and standard deviation (SD) of three replicates were used to express data. Different letters represent the statistical significant different at 95 % confident interval (p < 0.05) . T1= standard Guillard F/2 medium; T2 = modified Guillard F/2 medium supplemented with 0.05 g L⁻¹ sodium bicarbonate; T3 = modified Guillard F/2 medium with a 50% reduction in nitrogen. [file peerj-13-20098-s015.docx]

**Table 2** Total phenolic content and antioxidant activity content in crude diatom beta-glucan extracted samples, *C. muelleri*

| **Extract analysis** | **T1** | **T2** | **T3** |
| --- | --- | --- | --- |
| Total phenolic (μg/mg of GAE) | 14.91± 0.97^b^ | 4.44±0.11^a^ | 4.16±0.17^a^ |
| ABTS scavenging of 2 mg/ml extract | 89.21±1.71^b^ | 10.66±0.38^a^ | 94.59±0.04^c^ |
| DPPH scavenging of 10 mg/ml extract | 22.47±1.23^a^ | 23.34±0.67^a^ | 65.33±2.90^b^ |
| Reducing power (mg ascorbic acid) | 49.44±4.49^a^ | 47.61±5.45^a^ | 53.23±6.61^a^ |

The difference letters mean statistically significant at 95% confident interval (*p* < 0.05)

**Row Data**

| **Total phenolic (μg/mg of GAE)** | **T1** | **T2** | **T3** |
| --- | --- | --- | --- |
| **R1** | 14.71 | 4.43 | 4.31 |
| **R2** | 14.06 | 4.34 | 4.21 |
| **R3** | 15.97 | 4.55 | 3.97 |
| **Average** | 14.91 | 4.44 | 4.16 |
| **SD** | 0.97 | 0.11 | 0.17 |

| **ABTS scavenging of 2 mg/ml extract** | **T1** | **T2** | **T3** |
| --- | --- | --- | --- |
| **R1** | 88.63 | 11.07 | 94.62 |
| **R2** | 87.87 | 10.61 | 94.54 |
| **R3** | 91.13 | 10.31 | 94.62 |
| **average** | 89.21 | 10.66 | 94.59 |
| **SD** | 1.71 | 0.38 | 0.04 |

| **DPPH scavenging of 10 mg/ml extract** | **T1** | **T2** | **T3** |
| --- | --- | --- | --- |
| **R1** | 23.10 | 22.77 | 66.26 |
| **R2** | 23.26 | 23.18 | 62.08 |
| **R3** | 21.05 | 24.08 | 67.65 |
| **Average** | 22.47 | 23.34 | 65.33 |
| **SD** | 1.23 | 0.67 | 2.90 |

| **Reducing power (mg ascorbic acid)** | **T1** | **T2** | **T3** |
| --- | --- | --- | --- |
| **R1** | 54.29 | 51.27 | 57.31 |
| **R2** | 48.61 | 50.21 | 45.60 |
| **R3** | 45.42 | 41.34 | 56.77 |
| **Average** | 49.44 | 47.61 | 53.23 |
| **SD** | 4.49 | 5.45 | 6.61 |
