# Supplementary material for: Biochemical composition, β-glucan and phenolic content of a marine diatom Chaetoceros muelleri cultivated in Guillard’s modified medium
Source: PeerJ. 2025 Sep 30;13:e20098. doi: 10.7717/peerj.20098 (PMC12493710; doi:10.7717/peerj.20098)
Supplement: Supplemental Information 16 — The mean and standard deviation (SD) of three replicates were used to express data. Different letters represent the statistical significant different at 95 % confident interval (p < 0.05) . T1= standard Guillard F/2 medium; T2 = modified Guillard F/2 medium supplemented with 0.05 g L⁻¹ sodium bicarbonate; T3 = modified Guillard F/2 medium with a 50% reduction in nitrogen. [file peerj-13-20098-s016.docx]

**Table 3** Glucan content in crude diatom beta glucan extracted, *C. muelleri,* assayed with the Megazyme assay kit.

|  | **T1** | **T2** | **T3** |
| --- | --- | --- | --- |
| α-glucan (% w/w) | 0.059±0.003^c^ | 0.054±0.002^b^ | 0.013±0.002^a^ |
| β-glucan (% w/w) | 2.92±0.30^a^ | 11.59±0.18^b^ | 79.45±1.40^c^ |
| Total glucan (% w/w) | 2.98±0.30^a^ | 11.64±0.18^b^ | 79.47±1.40^c^ |

The mean and standard deviation (SD) of three replicates is used to express data. When employing one-way ANOVA (P< 0.05), different letters represent the statistical comparisons between groups.

**Row Data**

| **α-glucan (%w/w)** | **T1** | **T2** | **T3** |
| --- | --- | --- | --- |
| **R1** | 0.056 | 0.056 | 0.011 |
| **R2** | 0.062 | 0.054 | 0.014 |
| **R3** | 0.059 | 0.052 | 0.014 |
| **mean (%w/w)** | 0.059 | 0.054 | 0.013 |
| **SD** | 0.003 | 0.002 | 0.002 |

| **β-glucan (%w/w)** | **T1** | **T2** | **T3** |
| --- | --- | --- | --- |
| **R1** | 3.10 | 11.69 | 78.39 |
| **R2** | 3.10 | 11.38 | 78.92 |
| **R3** | 2.58 | 11.69 | 81.04 |
| **mean (%w/w)** | 2.92 | 11.59 | 79.45 |
| **SD** | 0.30 | 0.18 | 1.40 |

| **Total-glucan (%w/w)** | **T1** | **T2** | **T3** |
| --- | --- | --- | --- |
| **R1** | 3.15 | 11.75 | 78.40 |
| **R2** | 3.16 | 11.43 | 78.94 |
| **R3** | 2.64 | 11.75 | 81.06 |
| **mean (%w/w)** | 2.98 | 11.64 | 79.47 |
| **SD** | 0.30 | 0.18 | 1.40 |
