# Supplementary material for: Biochemical composition, β-glucan and phenolic content of a marine diatom Chaetoceros muelleri cultivated in Guillard’s modified medium
Source: PeerJ. 2025 Sep 30;13:e20098. doi: 10.7717/peerj.20098 (PMC12493710; doi:10.7717/peerj.20098)
Supplement: Supplemental Information 17 [file peerj-13-20098-s017.pdf]

Table 3 Glucan content in crude diatom beta glucan extracted, *C. muelleri*, assayed with the Megazyme assay kit.

	T1	T2	T3
α-glucan (% w/w)	0.059±0.003[c]	0.054±0.002[b]	0.013±0.002[a]
β-glucan (% w/w)	2.92±0.30[a]	11.59±0.18[b]	79.45±1.40[c]
Total glucan (% w/w)	2.98±0.30[a]	11.64±0.18[b]	79.47±1.40[c]

The mean and standard deviation (SD) of three replicates is used to express data. When employing one-way ANOVA ($P< 0.05$), different letters represent the statistical comparisons between groups.

Row Data

**α-glucan (%w/w)**	**T1**	**T2**	**T3**
**R1**	0.056	0.056	0.011
**R2**	0.062	0.054	0.014
**R3**	0.059	0.052	0.014
**mean (%w/w)**	0.059	0.054	0.013
**SD**	0.003	0.002	0.002

**β-glucan (%w/w)**	**T1**	**T2**	**T3**
**R1**	3.10	11.69	78.39
**R2**	3.10	11.38	78.92
**R3**	2.58	11.69	81.04
**mean (%w/w)**	2.92	11.59	79.45
**SD**	0.30	0.18	1.40

**Total-glucan (%w/w)**	**T1**	**T2**	**T3**
**R1**	3.15	11.75	78.40
**R2**	3.16	11.43	78.94
**R3**	2.64	11.75	81.06
**mean (%w/w)**	2.98	11.64	79.47
**SD**	0.30	0.18	1.40
